# Structural Properties and Stability of Proteins in Dihydrolevoglucosenone/Water Mixtures

**DOI:** 10.1021/acs.jpcb.5c02271

**Published:** 2025-07-28

**Authors:** Antonia Intze, Raffaella Polito, Maria Eleonora Temperini, Alessio Incocciati, Chiara Cappelletti, Sofia Botta, Michele Ortolani, Valeria Giliberti, Roberta Piacentini

**Affiliations:** † Center for Life Nano- & Neuro-science, Istituto Italiano di Tecnologia (IIT), Viale Regina Elena 295, 00161 Rome, Italy; ‡ Department of Biochemical Sciences “Alessandro Rossi Fanelli”, 9311 Sapienza University of Rome, Piazzale Aldo Moro 5, 00185 Rome, Italy; § Department of Physics, 9311 Sapienza University of Rome, Piazzale Aldo Moro 5, 00185 Rome, Italy; ∥ Institute for Photonics and Nanotechnologies IFN-CNR, Via del Fosso del Cavaliere, 100, 00133 Rome, Italy

## Abstract

Dihydrolevoglucosenone (DHL) shows great promise as an alternative to conventional toxic organic solvents widely used for industrial purposes. In this framework, evaluating the potential of DHL (commercially known as Cyrene) as a solvent for dissolving proteins is of great importance. Here, the effect of DHL/water mixtures on protein stability and solubility has been assessed. Several proteins, namely, hemoglobin, ferritin, ribonuclease, and albumin, were readily dissolved in buffer solutions containing up to 50–60% DHL and were stable at room temperature, as indicated by gel electrophoresis and matrix-assisted laser desorption/ionization analysis. Turbidimetry assays were performed in order to assess the solubility limitations derived from DHL/water mixtures. Finally, protein secondary structures in such mixtures, investigated by attenuated total reflectance Fourier-transform infrared spectroscopy, were found to be comparable to those obtained in phosphate buffer up to 50% DHL/water, with small spectral changes in the case of ribonuclease. DHL/water mixtures may thus represent highly convenient solvents for studies of protein chemistry.

## Graphical abstract

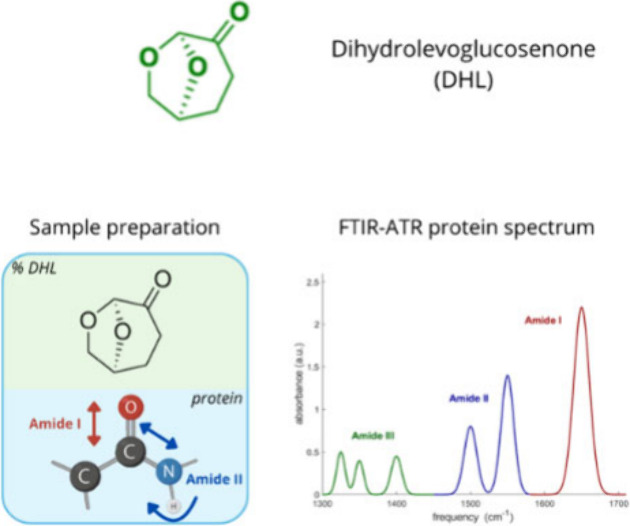

## 1 Introduction

Dihydrolevoglucosenone (DHL), commercially known as Cyrene, is a bicyclic, chiral, dipolar aprotic molecule that is obtained from a combination of chemical processes starting from biomass (mainly cellulose) that ultimately yield pure DHL.^1^

Recent studies^2–6^ have highlighted the advantages of using DHL in “green” organic chemistry, noting its biodegradability, non-mutagenic and non-toxic nature, high water miscibility, and safety in end-of-life disposal. So far, the properties of DHL have been investigated in the context of several key organic reactions,^7–10^ and in industrial separation processes, such as aromatic and aliphatic separations.

When mixed with water, DHL forms a geminal diol. Such a diol behaves as a hydrotrope: the two additional proton donor groups increase its overall hydrogen-bonding capacity, marking the DHL/water solvent system as amphiphilic.

In this framework, DHL/water mixtures provide a continuum of solvents characterized by adjustable polarity, depending on the DHL-to-water ratio. As such, many organic substances (e.g., pharma products, poorly soluble substrates, etc.) exhibit higher solubility in DHL/water mixtures with respect to aqueous buffers, thus increasing products concentration in the solution phase. At present, however, no data are available on the use of DHL in biochemistry and protein chemistry in particular, despite the high potential of such a solvent in solubilizing hydrophobic substrates or protein ligands in enzyme reactions, assessing biocatalysis, and optimizing antioxidant compounds.^11,12^ Thus far, as a first approach to the use of DHL in biochemical processes, an investigation of protein solubility and stability has been carried out in this work.

Studying the protein secondary structure in DHL/water mixtures and comparing it to that in water is essential for evaluating whether proteins maintain their native conformation. In this context, Fourier-transform infrared (FTIR) spectroscopy in the mid-IR range is a well-established tool for studying the conformation of proteins and its modification in either liquid or dry conditions.^13,14^ This versatile technique requires minimal sample preparation and provides insights into protein secondary structure by analyzing the amide I band, which falls within the 1600–1700 cm^–1^ range. This band primarily originates from the coupled vibrational modes of C=O stretching, with a minor contribution from the out-of-phase CN stretching, of each peptide unit within the protein polypeptide chain. Deconvolution of the amide I band allows for the quantification of the relative amounts of protein secondary structure components, such as α-helix, β-sheet, turn, and disordered conformation.^15,16^ FTIR spectroscopy operated in attenuated total reflection (ATR) mode is a surface-sensitive technique that provides higher sensitivity to protein conformation in hydrated or dry thin films compared to conventional transmission FTIR.^17,18^ Here, we performed ATR-FTIR spectroscopy to investigate the secondary structure of proteins in DHL/water mixtures and to assess the suitability of DHL as a protein solvent.

## 2 Materials and Methods

### 2.1 Chemicals and Reagents

Dihydrolevoglucosenone (DHL) was purchased from Sigma-Aldrich, as were bovine serum albumin (BSA), ribonuclease (RNase), and hemoglobin (Hb) proteins. Human ferritin (HFt) was expressed and purified according to the protocol found in Incocciati et al.^19^ All proteins were dissolved in 20 mM sodium phosphate buffer, pH 7.2, and then mixed with DHL at different concentrations.

### 2.2 Turbidimetry Measurements

The turbidimetric assay involved a time-course acquisition of absorbance at a fixed wavelength (600 nm) of a protein dissolved in various DHL/water mixtures. Measurements were performed using a Jasco V-750 UV–visible/NIR spectrophotometer (JASCO) with bandwidth set at 2 nm over a period of 1200 s (20 min) and *T* = 25 °C. Protein concentration was 25 mg/mL for BSA, 2.5 mg/mL for HFt, and 2 mg/mL for RNase. The assay was configured with a control measurement to establish a baseline for a favorable protein solubility. This control was represented by the trace obtained at 0% DHL (Figure 1, gray dashed lines).

### 2.3 Gel Electrophoresis Assays

Native electrophoresis analysis was carried out on 4% to 15% non-denaturing acrylamide gel (Mini-PROTEAN TGX stain-free, Bio-Rad) using Tris/Gly as a running buffer. Electrophoresis was performed at room temperature for 30–40 min at a constant voltage of 150–200 V using a Mini-Protean tetra-cell electrophoresis apparatus (Bio-Rad). Sodium dodecyl-sulfate polyacrylamide gel electrophoresis (SDS-PAGE) was carried out on 12% non-denaturing acrylamide gel (12% Mini-PROTEAN TGX Stain-Free) using Tris/Gly/SDS as a running buffer. Electrophoresis was performed under the same conditions as those used for the native.

### 2.4 MALDI-TOF MS Measurements

We compared the matrix-assisted laser desorption/ionization time-of-flight (MALDI-TOF) spectra of HFt dissolved in 50% DHL solution with those of HFt in buffer. Analysis was performed on freshly prepared samples, as well as on samples incubated in solution for 24 h and 1 week. Treated and untreated samples were analyzed by a MALDI ToFToF platform (ultrafleXtreme, Bruker), equipped with a smartbeam-II laser, in linear and positive mode, in a 5–20 kDa mass range. For these analyses, sinapic acid was utilized as a matrix, in a 1:1 (sample:matrix) ratio.

### 2.5 Fluorescence Quenching Measurements

We performed fluorescence signal acquisitions of BSA (1 mg/mL in distilled water) with the addition of curcumin dissolved in ethanol (EtOH) or a solution of EtOH/DHL 50%. Acquisitions were performed using a RF-6000 spectrofluorophotometer (Shimadzu). The excitation wavelength was set to 280 nm, with excitation and emission bandwidths of 1.5 and 3.0 nm, respectively. The concentration of BSA was 1 mg/mL in pure water. Then, the curcumin solution (pure EtOH or EtOH/DHL 50%) was successively titrated to the quartz cuvette containing 2 mL of BSA (the curcumin concentrations changed from 9 to 150 μM).

### 2.6 ATR-FTIR Measurements

Hb, BSA, RNase, and HFt proteins dissolved in a phosphate buffer solution and 50% DHL were studied by ATR-FTIR spectroscopy. ATR-FTIR spectra were acquired in liquid solution using a VERTEX 70v spectrometer (Bruker Optics GmbH) equipped with a N_2_ cooled mercury–cadmium-telluride detector from Infrared Associates Inc. The interferometer was purged with dry air from an FTIR purge-gas generator (Parker-Balston) to remove the IR absorption of atmospheric CO_2_ and water vapor. To collect spectra, a 50 μL aliquot of the solvent (either phosphate buffer alone or a 50% DHL/phosphate buffer mixture), or the protein in the solvent was cast on the center of the 45° germanium (Ge) crystal. The acquisition protocol was first to collect a spectrum of the solvent and then of the protein in the solvent. The spectrum of the clean Ge reference was acquired twice for each measurement set: first before the addition of the solvent and then after the addition of the protein in the solvent solution. Spectra were recorded in the range from 700 to 10,000 cm^–1^ with a spectral resolution of 2 cm^–1^, an aperture of 2 mm, and averaging 128 scans. Protein concentration was 40 mg/mL for BSA, 20 mg/mL for HFt, 9.5 mg/mL for Hb, and 34 mg/mL for RNase.

### 2.7 Infrared Spectra Analysis

The ATR-FTIR spectra shown in Figure 3 were baseline corrected, smoothed with a spline algorithm, and normalized by IgorPro 6.22A. To determine the relative amount of secondary structure components, the amide I band was fitted by Standard MATLAB (The Mathwork inc.) based least-squares fitting algorithm in the 1600–1700 cm^–1^ spectral range in terms of Gaussian-shaped secondary structure contributions using 3/5 Gaussian curves. Second-order derivatives of the ATR-FTIR spectra determined the number and peak positions of the Gaussian line shapes. The amide II band was fitted with one component centered at 1580 cm^–1^, with intensity and full width at half-maximum (FWHM) as the free parameters. The amide I band was deconvolved using 3/5 Gaussian components centered at 1620, 1635, 1655, 1670, and 1678 cm^–1^, ranging ±3 cm^–1^, of identical FWHM equal to 14 ± 2 cm^–1^ and intensity as the free parameter. The Gaussian line shapes used to deconvolve the amide I band are assigned to different secondary structure components of the protein: 1613–1637 and 1683–1700 cm^–1^ are ascribed to β-sheet, 1638–1648 cm^–1^ to disordered, 1652–1664 cm^–1^ to α-helix, and 1662–1686 cm^–1^ to β-turn.^20^

## 3 Results and Discussion

All proteins object of the present research were found to be fully soluble in DHL/phosphate buffer up to about 50% DHL/water (V/V) mixtures in spectrophotometric scattering measurements. A turbidimetric assay was performed to monitor changes in the optical density of bovine serum albumin (BSA), human ferritin (HFt), and ribonuclease (RNase) from bovine pancreatic proteins in mixed DHL/aqueous solutions at varying concentrations. The results are reported in Figure 1.

**Figure 1:**
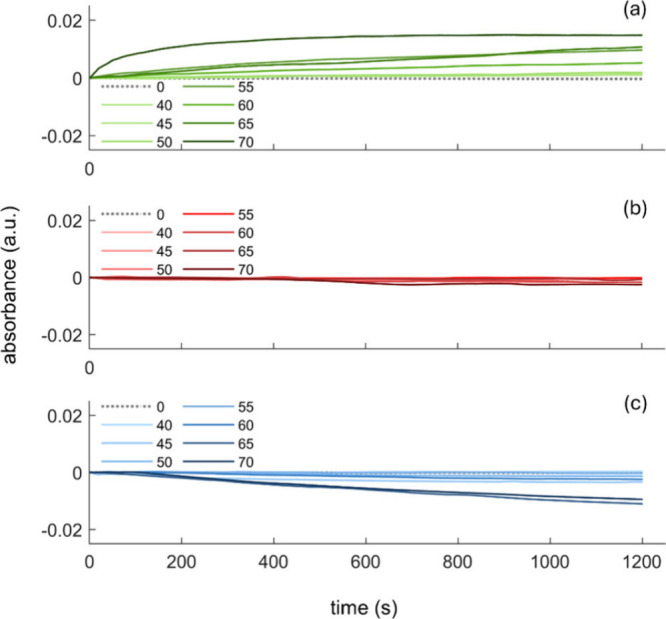
Time-course absorbance measurements of protein precipitation in DHL/buffer solutions. Solution mixtures of (a) BSA, (b) HFt, and (c) RNase proteins in phosphate buffer containing DHL concentrations ranging from 40% to 70% were recorded at a fixed wavelength (600 nm) for 1200 s (20 min) and *T* = 25 °C. Protein concentrations for each measurement were 25 mg/mL (BSA), 2.5 mg/mL (HFt), and 2 mg/mL (RNase).

For BSA, the absorbance increased at DHL concentrations of >50%, indicating enhanced protein aggregation under these conditions. In the case of HFt, no significant changes in absorbance were observed for DHL concentrations up to 70%, indicating a lack of aggregation or other measurable structural alterations under these conditions. In contrast, RNase showed a decrease in absorbance at DHL concentrations exceeding 60%. This trend may indicate either the dissolution of formed aggregates, leading to a clearer solution, or sedimentation of larger, denser particles at the bottom of the cuvette, reducing the apparent turbidity. Alternatively, interactions between DHL and the secondary structure of RNase might alter the refractive index of the particles, influencing the turbidimetric signal.

Gel electrophoresis assays under both native and denaturing conditions indicated virtually no changes in electrophoretic migration, ascribable to protein degradation or chemical modification of exposed amino groups. The results, presented in the Supporting Information (), demonstrate that the molecular weights of the proteins under study (BSA and HFt), incubated with varying concentrations of DHL (10%, 20%, 30%, and 50%) for different incubation times (0 and 24 h), remain unchanged compared to standard conditions. At longer times (1 week at 25 °C), however, in the case of HFt, a distinct peak at −128 ± 3 u.m.a. was observed in the matrix-assisted laser desorption/ionization time-of-flight mass spectrometry (MALDI-TOF MS) experiment (). Results show that up to 24 h incubation at 25 °C, the abundance of molecules corresponding to the *m*/*z* ratio of the HFt monomer (typical doublet at 21235 and 21367 Da) is maintained. A second peak arising at 21,112 Da (−128.13 Da concerning the first HFt peak) is observed, possibly due to N-terminal cleavage induced by the presence of DHL.

To evaluate whether DHL might hamper molecular binding processes, we performed a fluorescence quenching assay to quantify the interaction between BSA and curcumin across a range of curcumin concentrations. Curcumin, a naturally derived polyphenol, is broadly employed in food and medicine due to its characteristic taste and health benefits.^21^ However, its extremely poor water solubility and susceptibility to degradation from light, heat, and pH variations severely limit its use in nutraceuticals and pharmaceuticals.^22^ Complexing with food proteins has proven to be an effective strategy for enhancing curcumin’s water solubility, stability, and overall bioactivity.^23,24^ Recent research indicates that curcumin forms BSA–curcumin nanoparticles upon interaction with BSA, thereby improving its oral bioavailability and stability.^25,26^ Consequently, we opted to perform a spectroscopic analysis to observe the formation of the BSA–curcumin complex with curcumin dissolved in a 50% DHL solution. BSA possesses endogenous fluorescent properties attributed to its tryptophan, tyrosine, and phenylalanine residues.^27,28^ Fluorescence spectroscopy is an effective method for investigating interactions between small molecules and proteins. Changes in a protein’s intrinsic fluorescence can elucidate the binding mechanism between ligands and proteins. Therefore, we applied this strategy to evaluate the binding process between curcumin and BSA. As Figure 2a,b illustrates, the gradual addition of curcumin led to a consistent drop in BSA’s fluorescence intensity, alongside a noticeable blue shift (from 340 to 310 nm). This finding suggests that curcumin binds with BSA, forming a curcumin–BSA complex, which in turn alters the microenvironment of the fluorescent chromophore of the protein. For a more detailed understanding of the binding mechanism between BSA and curcumin, the Stern–Volmer equation was employed to analyze the fluorescence data:^29^

$$\frac{F_{0}}{F}=1+K_{\mathrm{SV}}\left[C\right]=1+K_{Q}\tau_{0}\left[C\right]$$

In this equation, *F*_0_ and *F* represent the fluorescence intensities of BSA in the absence and presence of curcumin, respectively, [C] is the concentration of curcumin, and *K*_SV_ is the Stern–Volmer dynamic quenching constant, which reflects the degree of the quenching process. *K*_Q_ and τ_0_ denote the bimolecular quenching rate constant and the fluorescence lifetime of the biopolymer, respectively. The Stern–Volmer plots for BSA quenched by various concentrations of curcumin in ethanol (EtOH) and EtOH/50% DHL exhibited good linear fits (*R* ^2^ = 0.92 and 0.97, respectively), as reported in Figure 2c. The calculated *K*_SV_ values were 1.2576 × 10^5^ and 4.1625 × 10^5^ M^–1^, respectively. These values correspond to *K*_Q_ values on the order of 10^14^ M^–1^ s^–1^ (assuming a τ_0_ value on the order of nanoseconds). Given that the diffusion-limited rate constant (the upper limit for *K*_Q_ in aqueous solution at room temperature) is typically on the order of 1 × 10^9^–10^10^ M^–1^ s^–1^, these results strongly suggest that the quenching between BSA and curcumin is predominantly a static quenching process. This implies that a stable complex readily forms between the fluorophore and the quencher under both solvent conditions.

**Figure 2:**
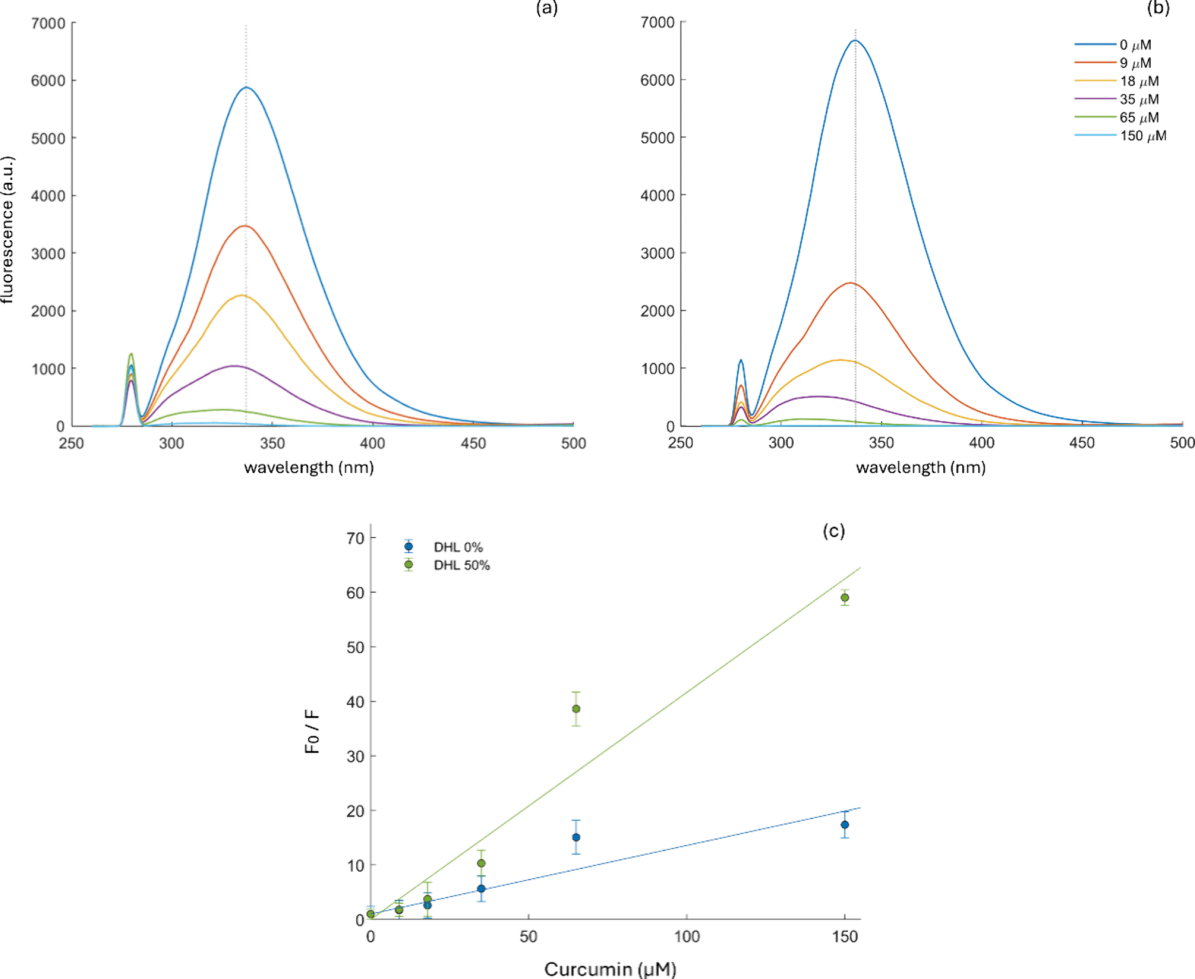
Effect of curcumin, dissolved in pure EtOH (panel a) and in 50% EtOH/DHL (panel b), on the fluorescence emission signals of chromophores in BSA solution (1 mg/mL in pure water). Panel c represents the Stern–Volmer plots for the fluorescence quenching of BSA by curcumin in the two different conditions.

In order to evaluate the effect of DHL on protein secondary structures, ATR-FTIR measurements were carried out.^17,18,30^ In all measurements, accurate subtraction of the DHL/buffer baseline was achieved according to the recorded spectra of DHL in sodium phosphate buffer 20 mM, pH 7.2, at different DHL concentrations (Figure 3), and results were in good agreement with previous measurements.^31^ It should be noted that DHL displays a convenient spectral window in the amide I and II regions (1700–1500 cm^–1^), thus flattening the water contribution at 1645 cm^–1^ and offering a better signal-to-noise ratio for proteins even at protein concentrations lower than those typically used in plain H_2_O-based buffers.

**Figure 3:**
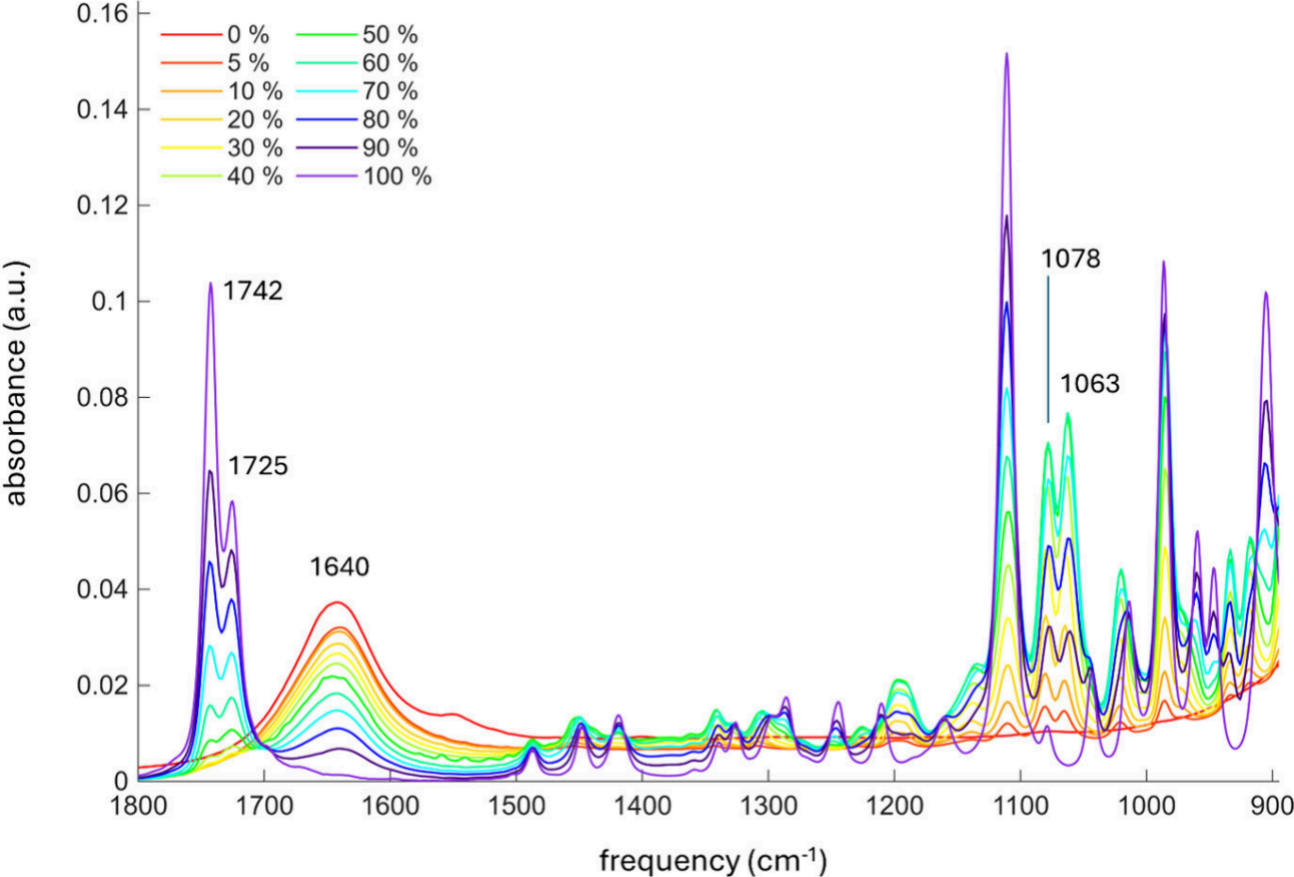
ATR-FTIR spectra of DHL/sodium phosphate buffer mixtures in different % of DHL.

Figure 3 shows the ATR-FTIR spectra of pure DHL and the DHL/sodium phosphate buffer mixtures. Comparing the spectra, it is evident that an increase in DHL content results in variations in the relative intensities of the carbonyl stretching bands at 1725 and 1742 cm^–1^, which correspond to the formation of DHL oligomers and monomeric DHL, respectively.^31^ The bands at 1725 and 1742 cm^–1^ reached equal intensity in a 70% DHL/buffer mixture. Additionally, progressive variations were detected in the C–C skeletal vibrational bands in the 900–1000 cm^–1^ region and the relative intensities of the symmetric and asymmetric OH stretching modes of the geminal diol at 1078 and 1063 cm^–1^, respectively, with an increasing DHL content. Finally, as expected, the band centered at 1645 cm^–1^, associated with the bending vibrational mode of the OH group in water molecules, increased linearly with the buffer content.

To assess whether proteins undergo changes in their native secondary structure when dissolved in DHL, ATR-FTIR spectra were measured in the amide I and amide II band regions for BSA, HFt, Hb, and RNase dissolved in phosphate buffer and compared to those dissolved in 50% DHL/buffer mixtures (Figure 4). Samples were prepared under conditions suitable for a technique that requires proteins to be sufficiently concentrated. Therefore, the BSA concentration was set to 40 mg/mL, HFt to 20 mg/mL, Hb to 9.5 mg/mL, and RNase to 34 mg/mL. Given the well-established sensitivity of the amide I vibration to protein secondary structure,^13^ particular attention was given to modifications in the amide I band line shape. The amide II band is generally less responsive to changes in protein secondary structure^32^ and therefore the modifications in this spectral region are not addressed in the current discussion. Notably, the amide I band line shapes of all investigated proteins were quite similar in buffer-only and in the 50% DHL/buffer mixture. It is important to note that liquid water strongly absorbs at 1645 cm^–1^ (bending of H–O–H), and imperfect subtraction of its contribution may result in minor differences in the absorbance spectra of proteins. Thus, the small variations observed around 1640 cm^–1^ for BSA and RNase (Figure 4a,d) are likely attributed to the displacement of solvent by protein molecules on the Ge crystal or differences in the hydration levels of the proteins.

**Figure 4:**
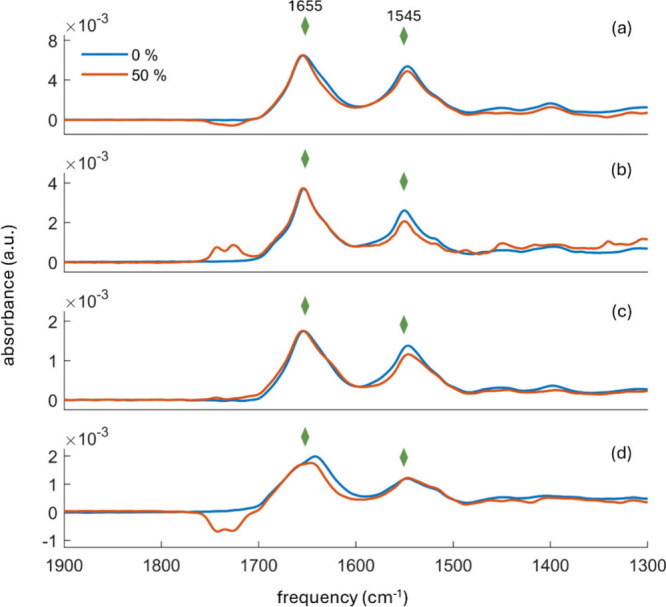
ATR-FTIR spectra of (a) BSA, (b) HFt, (c) Hb, and (d) RNase in buffer alone (blue line) and 50% DHL/buffer solvent (red line). The green rhomboid symbols correspond to the frequencies 1655 and 1545 cm^–1^, assigned to α-helices in the amide I and amide II spectral range, respectively. Spectra are normalized at 1655 cm^–1^. The DHL spectral contributions in the 1710–1760 cm^–1^ range are due to imperfect solvent subtraction.

To further evaluate potential changes in the secondary structure of proteins dissolved in a 50% DHL/buffer mixture, second-order derivatives of the spectra were calculated and compared with those of proteins dissolved in buffer alone. In particular, Figure 5 shows the second derivative analysis of both amide I and II bands, whereas complete deconvolution of the amide I spectral envelope into Gaussian contributions is reported in Figure 6 for all studied proteins dissolved in buffer and 50% DHL/buffer mixtures. Modifications in the amide I band region reflect the overall stability of the protein secondary structures in different solvent environments. Specifically, the components centered around 1655 cm^–1^ correspond to α-helix structures, those at 1630 and 1690 cm^–1^ to β-sheets, those at 1645 cm^–1^ to disordered conformations, and those at 1680 cm^–1^ to turns.^30^ The α-helix component contributed most prominently to the second derivatives across BSA, HFt, and Hb, consistent with literature reports, which confirm a predominantly α-helical native structure for BSA, HFt, and Hb.^33–35^

**Figure 5:**
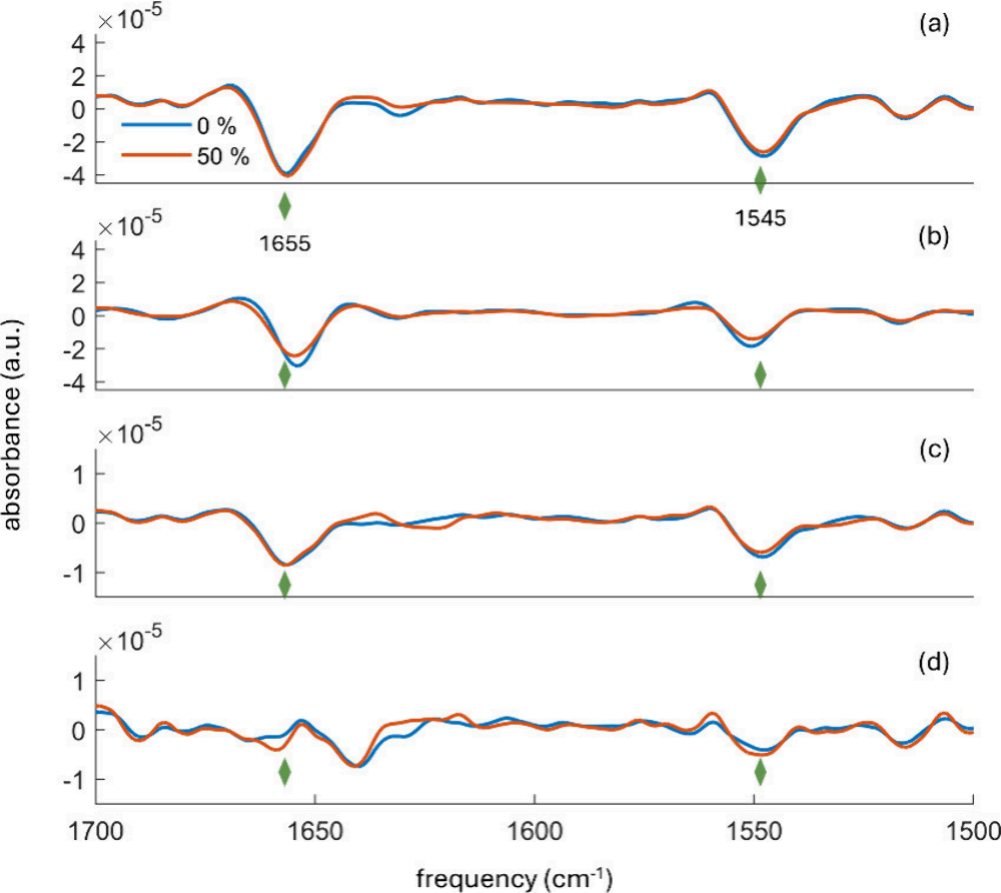
Second derivatives of ATR-FTIR spectra for (a) BSA, (b) HFt, (c) Hb, and (d) RNase in buffer (blue line) and 50% DHL/buffer (red line) solvent. The green rhomboid symbols correspond to the frequencies 1655 and 1545 cm^–1^, assigned to α-helices in the amide I and amide II spectral ranges, respectively.

Notably, BSA dissolved in the buffer-only environment showed a higher β-sheet contribution compared to BSA in 50% DHL/buffer mixture, while the α-helix contribution remained similar in both solvent environments (Figure 5a). Moreover, the second derivative curves of HFt in the buffer aligned well with those in the 50% DHL/buffer mixture with only slight variations in the α-helix component (Figure 5b). In the 50% DHL/buffer mixture, the α-helix content of Hb was quite similar to that in buffer-only, but a higher β-sheet contribution was observed (Figure 5c). Finally, RNase demonstrated an important contribution from disordered conformations compared to the other studied proteins, in agreement with literature reports.^36^ Also, in this case, there is a good agreement between RNase in buffer and in 50% DHL/buffer solvent, with minor differences observed in the α-helix and β-sheet regions (Figure 5d). For all studied proteins, no significant changes were detected in the turn region.

Protein denaturation is typically characterized by a significant decrease in α-helix content, along with an increase in β-sheet and turn components,^37^ leading to shifts in the maximum frequencies of the amide I and amide II bands toward higher wavenumbers.^38^ In this study, the minor variations observed in the α-helix and β-sheet regions across all protein samples (BSA, Hb, RNase, and HFt) did not indicate protein denaturation, as such changes were not accompanied by a substantial alteration in the turn region. Overall, the ATR-FTIR spectra and their second derivatives suggest that BSA, Hb, RNase, and HFt retained their native secondary structure in DHL/phosphate buffer mixtures up to DHL concentrations of 50%.

All spectra were further analyzed in the amide I band range in terms of Gaussian shaped secondary structures contributions by the least-squares method using 3/5 Gaussian curves plus a larger Gaussian as a baseline correction taking into account the contributions from amide II (Figure 6). Fitting parameters are given in Table 1. The standard MATLAB-based (The Mathwork inc.) least-squares fitting algorithm was used.

**Figure 6:**
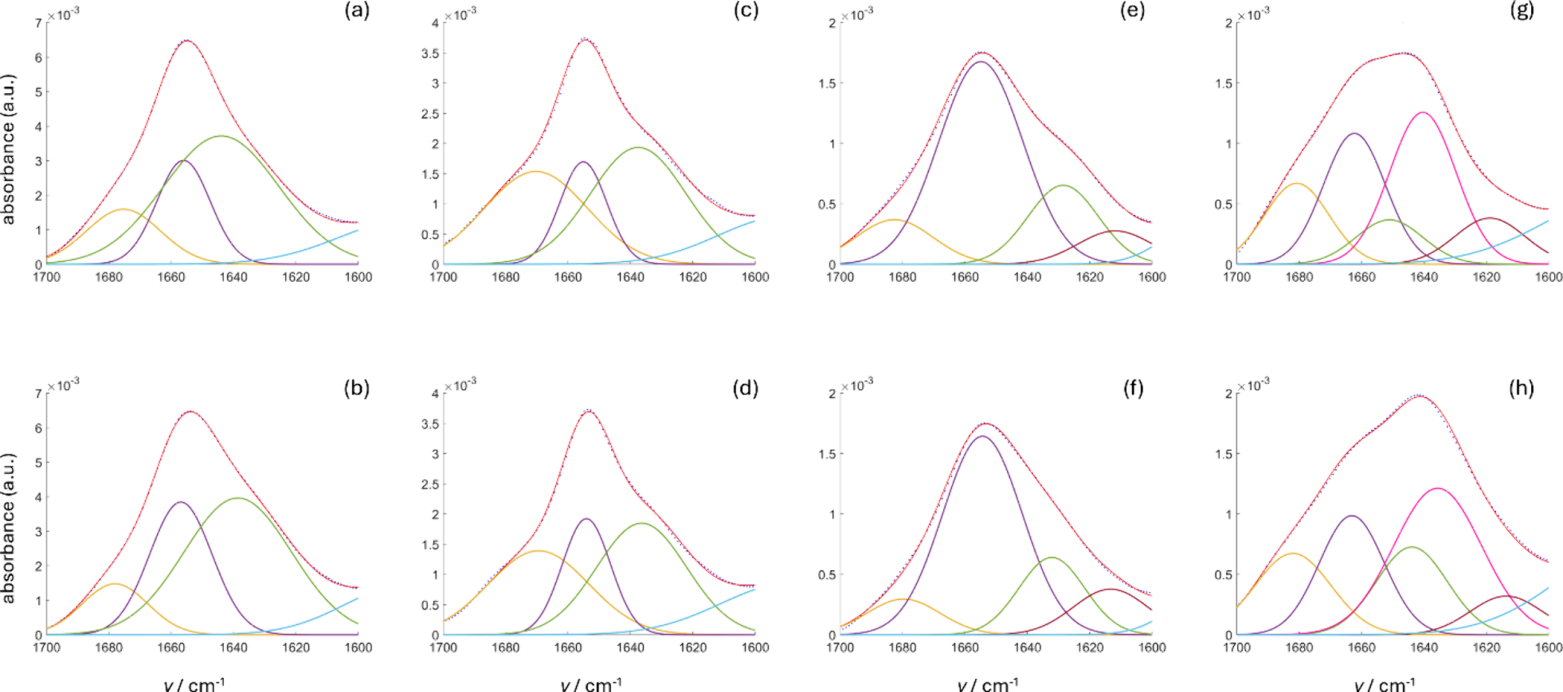
Gaussian fit of IR spectra in the amide I frequency range of proteins in different conditions: (a) BSA in 50% DHL/buffer mixture, (b) BSA in buffer, (c) HFt in 50% DHL/buffer mixture, (d) HFt in buffer, (e) Hb in 50% DHL/buffer mixture, (f) Hb in buffer, (g) RNase in 50% DHL/buffer mixture, and (h) RNase in buffer. Single Gaussian contributions of the amide I signal are in yellow (turn), purple (α-helix), pink (disordered), green and brown (β-sheet). The Gaussian curve in cyan centered at 1580 cm^–1^, accounts for the spectral contributions from amide II. The combination of all Gaussian curves (fitting curves represented by red lines) interpolates the experimental data (represented by blue dots).

**Table 1:** Curve Fitting Parameters for Amide I Spectrum

**Protein**	**Peak 1**	**Peak 2**	**Peak 3**	**Peak 4**	**Peak 5**
*solvent*	(% area) freq cm^–1^ (FWHM cm^–1^)	(% area) freq cm^–1^ (FWHM cm^–1^)	(% area) freq cm^–1^ (FWHM cm^–1^)	(% area) freq cm^–1^ (FWHM cm^–1^)	(% area) freq cm^–1^ (FWHM cm^–1^)
**BSA**	(16.7%) 1675 (12)		(22.7%) 1656 (9)	(60.6%) 1644 (19)	
*50% DHL*					
**BSA**	(12.7%) 1678 (11)		(31.5%) 1657 (10)	(55.8%) 1639 (17)	
*buffer*					
**HFt**	(19.5%) 1679 (13)		(50.8%) 1654 (11)	(12.4%) 1632 (8)	(17.3%) 1617 (13)
*50% DHL*					
**HFt**	(21.4%) 1675 (12)		(46.2%) 1653 (9)	(15.3%) 1634 (8)	(17.1%) 1619 (13)
*buffer*					
**Hb**	(12.1%) 1683 (12)		(59.7%) 1655 (14)	(19.5%) 1629 (11)	(8.7%) 1612 (12)
*50% DHL*					
**Hb**	(9.7%) 1680 (12)		(58.9%) 1654 (13)	(18.8%) 1632 (11)	(12.6%) 1613 (12)
*buffer*					
**RNase**	(17.7%) 1681 (10)	(27.4%) 1662 (10)	(10.0%) 1651 (11)	(34.1%) 1640 (11)	(10.9%) 1619 (11)
*50% DHL*					
**RNase**	(17.3%) 1682 (12)	(21.5%) 1663 (10)	(17.4%) 1644 (11)	(36.0%) 1636 (14)	(7.8%) 1614 (12)
*buffer*					

Spectra were corrected by subtracting the solvent contribution (buffer or 50% V/V DHL/buffer) below 1600 cm^–1^ by a single large Gaussian centered at 1580 cm^–1^ of 110 ± 0.5 cm^–1^ full width at half-maximum (FWHM). Individual Gaussian contributions to the spectral envelope for each protein were fitted by using Gaussian curves of identical FWHM’s for each spectrum. Three to five curves were necessary in order to reproduce the observed amide I spectral line shape. Fitting curves grossly represent secondary structure contributions in the observed FTIR spectra.^10^

It should be noted also here that small differences around 1640 cm^–1^ between proteins in buffer or DHL/buffer mixtures can be attributed to non-optimal subtraction of liquid water signal or due to the protein hydration shell. The amide I spectral envelope analysis of all proteins (see Figure 6), as inferred by the “minimal” Gaussian deconvolution (no Fourier self-deconvolution or self-consistent methods were applied), with 0% and 50% DHL, indicates that peaks and line widths relative to major secondary structure contributions (see Table 1) are fundamentally unchanged. However, relative intensities of α-helix and β-sheet structures in BSA and RNase appear to increase by 5–10% at 50% DHL/buffer, possibly due to the binding of DHL to relevant protein pockets or partial dehydration of solvent exposed regions.

## 4 Conclusions

The primary objective of our study was to investigate the effects of DHL on both the solubility and secondary structure of proteins when used as a co-solvent in DHL/buffer mixtures at varying DHL concentrations.

Based on the results obtained from turbidimetric assays, we evaluated the solubility of the selected proteins (BSA, HFt, and RNase) under different DHL concentration conditions. Our findings indicate that these proteins maintain an acceptable level of solubility up to a DHL concentration of 50%.

Electrophoretic assays, performed under both denaturing and native conditions, showed no significant changes in the electrophoretic migration. This suggests that the proteins do not undergo noticeable denaturation or degradation upon exposure to DHL. Similarly, mass spectrometric analysis using MALDI-TOF revealed no substantial alterations in protein integrity, aside from a minor shift that may correspond to the DHL-induced cleavage of the N-terminal amino acids.

A fluorescence quenching assay was performed as a proof-of-concept for the well-established BSA–curcumin binding reaction. We conducted the assay by directly adding various amounts of curcumin, dissolved in either EtOH or a 50% EtOH/DHL mixture, into a quartz cuvette, containing a solution of BSA at a fixed concentration. The observed quenching effect, indicative of interactions between the protein and the fluorophore molecule, was comparable across both solutions. This suggests that the presence of DHL does not influence the binding affinity of curcumin for BSA.

To further assess the structural impact of DHL on protein conformation, we employed ATR-FTIR spectroscopy to analyze the secondary structure components of the selected proteins in both buffer and 50% DHL/buffer mixtures. Changes observed in the amide I band region provide insights into potential variations in the structural integrity under different solvent conditions.

Notably, slight modifications in the amide I spectral envelope were observed in the presence of 50% DHL/buffer mixtures, particularly for BSA and RNase. In the case of BSA, the spectrum in buffer solution exhibited a higher β-sheet content compared with the 50% DHL/buffer mixture, likely due to solvent displacement effects, whereas the α-helix fraction remained largely unchanged between the two conditions. Similarly, the second derivative spectra of HFt in buffer-only closely resembled those recorded in the 50% DHL/buffer mixture, with only minor deviations in the α-helix contribution. For Hb, the α-helix content showed little variation between the two solvent environments, whereas an increased β-sheet component was detected in the 50% DHL/buffer solution. In contrast, RNase exhibited a higher proportion of disordered structures compared to the other proteins, which aligns with findings reported in the literature.^36^ While the spectral profiles of RNase in buffer and in 50% DHL/buffer mixtures were largely comparable, subtle, yet meaningful differences emerged in the α-helix and β-sheet regions. Across all analyzed proteins, no significant alterations were detected in the spectral regions associated with turns. The overall results suggest that proteins largely preserve their native secondary structure and stability in DHL-containing solutions.

Further studies will be necessary to fully characterize the extent of these interactions and their implications for protein stability. Our findings provide significant insights with potential applications across diverse areas of protein science, for example, facilitating the solubilization of hydrophobic ligands or improving the extraction and purification of integral membrane proteins, which often present significant challenges due to their inherent low solubility in aqueous environments. Furthermore, the observed modification of protein secondary structure by DHL could be exploited in enzymatic reactions where product inhibition or toxicity is a concern, potentially shifting equilibria or altering substrate accessibility to enhance reaction efficiency and yield. The combination of biochemical assays and ATR-IR spectroscopy presented in this study underscores the utility of this approach for probing subtle, yet significant, changes in protein structure in response to co-solvent environments.

## Supplementary Material





## Notes

IR spectra data set (mean *N* = 3) collected for the research discussed in the present scientific paper (XLSX)
SDS-PAGE and native electrophoresis acquisition and MALDI-TOF spectra (PDF)
