# Supplementary material for: Structural Properties and Stability of Proteins in Dihydrolevoglucosenone/Water Mixtures
Source: J Phys Chem B. 2025 Jul 28;129(31):7875–83. doi: 10.1021/acs.jpcb.5c02271 (PMC12337083; doi:10.1021/acs.jpcb.5c02271)
Supplement: Supplementary file 2 [file jp5c02271_si_002.pdf]

Supporting information

Structural Properties And Stability Of Proteins In Dihydroxylevoglucosenone/Water Mixtures

Antonia Intze[1,2], Raffaella Polito[3,4], Maria Eleonora Temperini[3], Alessio Incocciati[2], Chiara Cappelletti[2], Sofia Botta[2], Michele Ortolani[1,3], Valeria Giliberti[1], and Roberta Piacentini[2].*

Antonia Intze (1,2) antonia.intze@uniroma1.it

Raffaella Polito (3,4) raffaella.polito@cnr.it

Maria Eleonora Temperini (3) mariaeleonora.temperini@uniroma1.it

Alessio Incocciati (2) alessio.incocciati@uniroma1.it

Chiara Cappelletti (2) chiara.cappelletti@uniroma1.it

Sofia Botta (2) sofia.botta@uniroma1.it

Michele Ortolani (1,3) michele.ortolani@uniroma1.it

Valeria Giliberti (1) valeria.giliberti@iit.it

Roberta Piacentini * (2) roberta.piacentini@uniroma1.it

(1) Center for Life Nano- & Neuro-science, Istituto Italiano di Tecnologia (IIT), Viale Regina Elena 295, 00161, Rome, Italy

(2) Department of Biochemical Sciences “Alessandro Rossi Fanelli”, Sapienza University of Rome, Piazzale Aldo Moro 5, 00185, Rome, Italy

(3) Department of Physics, Sapienza University of Rome, Piazzale Aldo Moro 5, 00185, Rome, Italy

(4) Institute for Photonics and Nanotechnologies IFN-CNR, Via del Fosso del Cavaliere, 100, 00133, Rome, Italy

Corresponding Author

* Roberta Piacentini: roberta.piacentini@uniroma1.it

SDS-PAGE and native electrophoresis

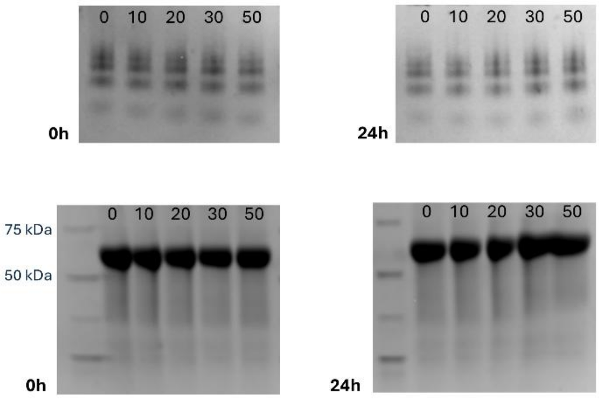

Figure S1. Electrophoresis gel of bovine serum albumin in solution with sodium phosphate buffer (20mM pH 7.2) and different concentrations of DHL (10, 20, 30, 50%) and incubated for 24 hours. Top panels show results in native conditions, bottom panels show results in denaturant conditions. Gels were dyed with Comassie.

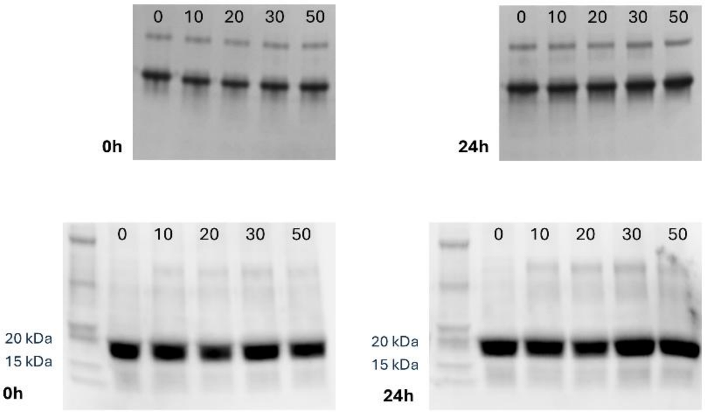

Figure S2. Electrophoresis gel of human H-ferritin (HFt) in solution with sodium phosphate buffer (20mM pH 7.2) and different concentrations of DHL (10, 20, 30, 50%) and incubated for 24 hours. Top panels show results in native conditions, bottom panels show results in denaturant conditions.

MALDI-TOF

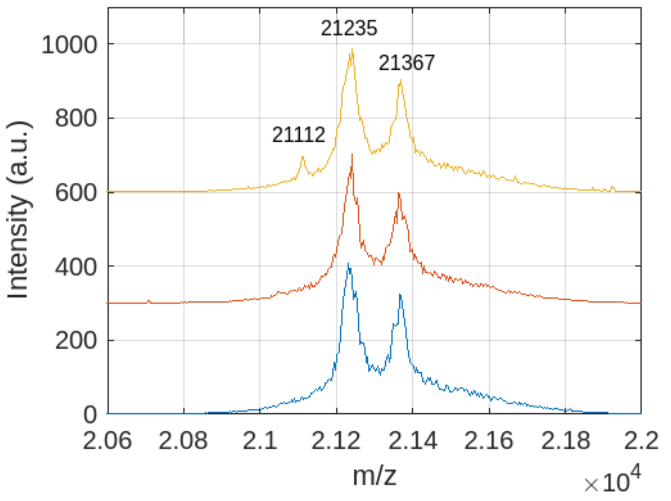

Figure S3. MALDI-TOF spectra of native HFt protein in buffer (blue) and with 50% DHL incubated for 24h (red) and one week (yellow).
